# Notch signal strength controls cell fate in the haemogenic endothelium

**DOI:** 10.1038/ncomms9510

**Published:** 2015-10-14

**Authors:** Leonor Gama-Norton, Eva Ferrando, Cristina Ruiz-Herguido, Zenhy Liu, Jordi Guiu, Abul B. M. M. K. Islam, Sung-Uk Lee, Minhong Yan, Cynthia J. Guidos, Nuria López-Bigas, Takahiro Maeda, Lluis Espinosa, Raphael Kopan, Anna Bigas

**Affiliations:** 1 Program in Cancer Research, Institut Hospital del Mar d'Investigacions Mèdiques (IMIM), Barcelona 08003, Spain; 2 Division of Developmental Biology, Department of Pediatrics, University of Cincinnati, Cincinnati, Ohio 45229-3026, USA; 3 Research Unit on Biomedical Informatics, Department of Experimental and Health Sciences, Universitat Pompeu Fabra, Barcelona 08003, Spain; 4 Department of Genetic Engineering and Biotechnology, University of Dhaka, Dhaka 1000, Bangladesh; 5 Department of Medicine, Harvard Medical School, Brigham and Women's Hospital, Boston, Massachusetts 02115, USA; 6 Department of Molecular Biology, Genentech, South San Francisco, California 94080, USA; 7 Program in Developmental and Stem Cell Biology, Department of Immunology, Hospital for Sick Children Research Institute, Toronto, Ontario, Canada M5G 0A4; 8 Institució Catalana de Recerca i Estudis Avançats (ICREA), Barcelona 08010, Spain

## Abstract

Acquisition of the arterial and haemogenic endothelium fates concurrently occur in the aorta–gonad–mesonephros (AGM) region prior to haematopoietic stem cell (HSC) generation. The arterial programme depends on Dll4 and the haemogenic endothelium/HSC on Jag1-mediated Notch1 signalling. How Notch1 distinguishes and executes these different programmes in response to particular ligands is poorly understood. By using two Notch1 activation trap mouse models with different sensitivity, here we show that arterial endothelial cells and HSCs originate from distinct precursors, characterized by different Notch1 signal strengths. Microarray analysis on AGM subpopulations demonstrates that the Jag1 ligand stimulates low Notch strength, inhibits the endothelial programme and is permissive for HSC specification. In the absence of Jag1, endothelial cells experience high Dll4-induced Notch activity and select the endothelial programme, thus precluding HSC formation. Interference with the Dll4 signal by ligand-specific blocking antibodies is sufficient to inhibit the endothelial programme and favour specification of the haematopoietic lineage.

## Abstract (web summary)

It is unclear how Notch1 signals regulate both the maintenance of the endothelial fate and the endothelial-to-hematopoietic transition in the embryonic aorta. Here the authors show that those cells in which Notch1 ligand Jag1 is out-competed by Dll4 remain endothelial, while higher Jag1 activity leads to generation of hematopoietic stem cells.

## Graphical abstract

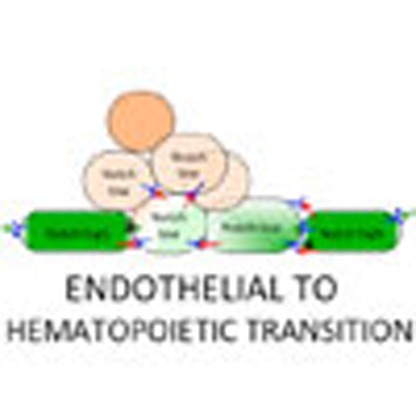

Haematopoietic stem cells (HSCs) are generated during embryonic life in the aorta–gonad–mesonephro (AGM) region^1^. This process requires gain of haematopoietic competence from cells displaying endothelial traits located in the embryonic aorta (also known as endothelial-to-haematopoietic transition (EHT)^2^,^3^,^4^) Recently, it has been demonstrated that the first molecular event in the EHT process requires the silencing of the endothelial programme^5^; however, the molecular signals governing the sequence of events to obtain a functional HSC are mainly unknown.

Notch1 signalling is indispensable for the specification of the arterial programme and the generation of HSCs^6^,^7^,^8^,^9^,^10^,^11^. Ligand specificity for each process has been suggested since deletion of Delta-like 4 (Dll4) results in strong arterial defects^12^,^13^, while Jagged1 (Jag1) deletion impairs definitive haematopoiesis^7^. The main structural difference between both types of ligands resides in the number of epidermal growth factor (EGF)-like repeats (6–8 for Delta and 16 for Jagged) and in the presence of C-rich domain in Jag1; however, ligand-mediated cleavage is thought to be a 'no memory' process in relation to the identity of the ligand involved^14^. Glycosylation of Notch by the fringe family of glycosyl-transferases^15^ was found to favour the association of Notch1 to Delta instead of Jagged ligands^16^, likely affecting Notch signal strength.

We have recently developed two mouse lines that trace cells that activate the Notch pathway and their descendants. Importantly, *N1IP::Cre*^*LO*^ is a low-sensitivity line that only traps cells experiencing high levels of Notch1 activation^17^, whereas *N1IP::Cre*^*HI*^ is high sensitive and traps cells experiencing both low and high levels of Notch activation^18^ (HI and LO designations reflect the differential sensitivity of these reporters defined here as the number of Notch intracellular domain (NICD) molecules released)^19^. We here demonstrate that, whereas N1IP::Cre^HI^ labels both haematopoietic and arterial cells, N1IP::Cre^LO^ specifically labels the arterial population, indicating that arterial and haematopoietic cells originate from different Notch-traceable populations. In addition, Jag1 restricts Notch activation in the haemogenic endothelium, which results in reduced expression of the endothelial gene programme and increased haematopoietic-specific transcription. Together, these results indicate that Jag1 is required to maintain the low Notch signal that is required for haematopoietic specification, whereas Dll4 secures the high Notch activity and the success of the arterial programme.

## Results

### Different Notch1 activity specifies haematopoietic and arterial fate

**Figure 1:**
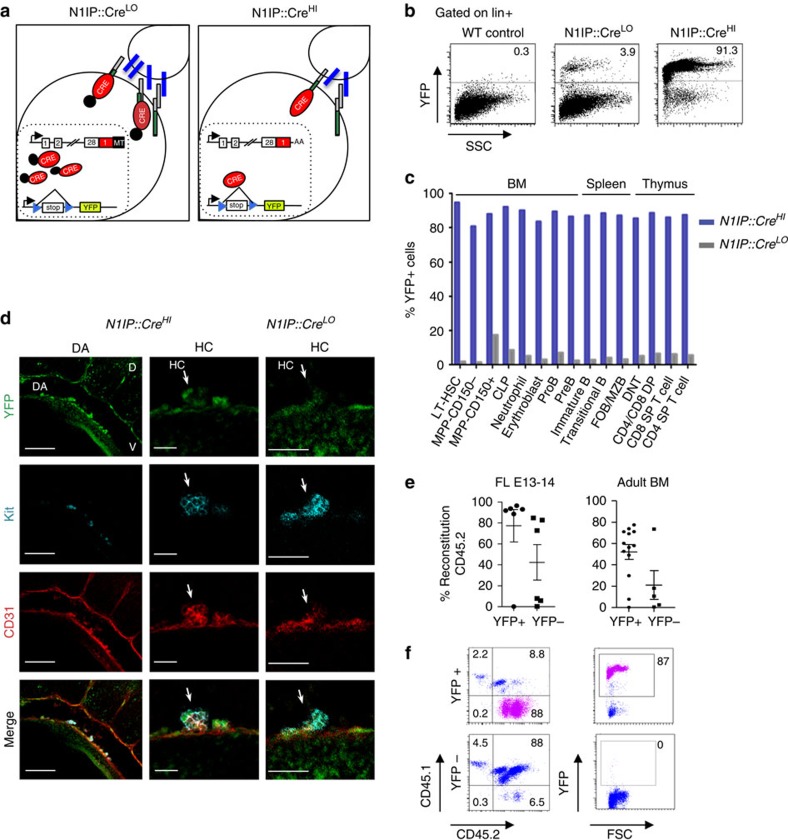
Haematopoietic and arterial specification requires different levels of Notch1 activity. (**a**) Schematic representation of Notch activation history mouse reporters by replacing the intracellular domain of mouse Notch1 with low sensitivity (N1IP::Cre^LO^) and high sensitivity (N1IP::Cre^HI^) Cre-recombinase. Reporter activation of N1IP::Cre^LO^ requires a high threshold of Notch activity, while N1IP::Cre^HI^ is induced in response to low or high Notch activity. (**b**) Flow cytometry analysis of peripheral blood of adult mice. Cells were stained with Lineage (lin) markers (CD3, B220, Gr1, Mac1 and Ter119) gated on lin+ cells. Numbers indicate the percentage of YPF+ cells. (**c**) Graph represents the percentage of YFP+ cells within haematopoietic cell types in the bone marrow (BM), spleen and thymus of N1IP::Cre^LO^ (grey bars) and N1IP::Cre^HI^ (blue bars) as detected using flow cytometry. (**d**) Representative confocal images of three-dimensional whole-mount immunostaining in N1IP::Cre^HI^ and N1IP::Cre^LO^ embryos (E10.5) detecting YFP (green), c-Kit (cyan) and CD31 (red). General view of the dorsal aorta (left panel) and details of haematopoietic cluster (right panels). White arrows indicate cluster structures. D, dorsal; DA, dorsal aorta, HC, haematopoietic cluster; V, ventral. Scale bars, 100 μm for DA, 25 μm for HC in N1IP::Cre^HI^ and 50 μm in N1IP::Cre^Low^. See also Supplementary Fig. 1. (**e**,**f**) Graphs show the percentage of reconstituted cells in animals transplanted with YFP+ and YFP− fractions of E13-14 fetal liver and BM at 4-month post-transplantation (**e**). Representative dot plots from analysis (**f**). Donor CD45.2 N1IP::Cre^HI^ cell fractions together with 500,000 supporting CD45.1 spleen cells were transplanted into CD45.1/CD45.2 chimeras.

Genetic studies have demonstrated that Notch1 is required for both haematopoietic and arterial specification^6^,^10^,^11^. Previously, we generated a genetic sensor of the Notch activation history by replacing the intracellular domain of mouse *Notch1* with the site-specific Cre-recombinase^17^ (Fig. 1a) and crossing these mice with the *ROSA*^*eYFP*^ reporters. In the double transgenic embryos (*N1IP::Cre*^*LO*^; *ROSA*^*eYFP*^), arterial endothelium including the dorsal aorta of the AGM region was detected as early as E10.5 at the time of HSC formation (Supplementary Fig. 1) and was more clear and intense after E11, when full arteries but no veins were labelled^17^. However, YFP+ cells among the haematopoietic lineages were barely detected in these mice (Fig. 1b) despite the fact that haematopoietic stem cell (HSC) development from the AGM endothelium also requires Notch1 signalling. These results indicated that YFP+ endothelial cells of the *N1IP::Cre*^*LO*^ AGM region are not the precursors of the definitive HSCs (YFP−) and strongly suggested that Notch activation in the haematopoietic lineage was insufficient to accumulate enough Cre molecules to rearrange the YFP reporter (as demonstrated in ref. 19).

To further investigate this possibility, we pursue for a strategy to trap cell lineages experiencing low levels of Notch activity. We found that removal of the tag from the Cre recombinase improved Cre activity, and consequently labelling efficiency (we refer to this transgene as *N1IP::Cre*^*HI*^)^19^. In contrast to that observed in the haematopoietic lineages of the *N1IP::Cre*^*LO*^ mice, we found a consistent YFP+ staining in the different haematopoietic organs and cell lineages of the *N1IP::Cre*^*HI*^;*ROSA*^*eYFP*^ mice (Fig. 1b,c). Comparative analysis of E10.5 *N1IP::Cre*^*HI*^ and *N1IP::Cre*^*LO*^ embryos using whole-mount immunostaining demonstrated that both lines contained YFP+ cells in the aortic endothelium (Supplementary Fig. 1), but only the *N1IP::Cre*^*HI*^ haematopoietic cluster cells (Kit+) were YFP+ (Fig. 1d). In addition, YFP+ cells isolated from the fetal liver or bone marrow of the *N1IP::Cre*^*HI*^;*ROSA*^*eYFP*^ reconstituted the haematopoietic system of lethally irradiated hosts (Fig. 1e,f). Thus, the *N1IP::Cre*^*HI*^ and *N1IP::Cre*^*LO*^ lines both labelled cell lineages that experience high levels of Notch activation (such as the arterial cells), but they differ in their capacity to label cells with a history of low Notch1 activity such is the case of the haematopoietic lineage. Moreover, these results indicate that definitive HSCs originate from endothelial precursors that have not experienced high Notch signal. Because Jag1 and Dll4 ligands are specifically required to promote the haematopoietic^7^ and the arterial^12^,^13^ fates, respectively, and different Notch signalling strength has previously been assigned to each ligand^20^, we propose that low (haematopoietic) and high (arterial) Notch activity observed in the embryonic AGM might be achieved by the differential use of either ligand.

### Jag1 and Dll4 distribution in the embryonic aortic cells

**Figure 2:**
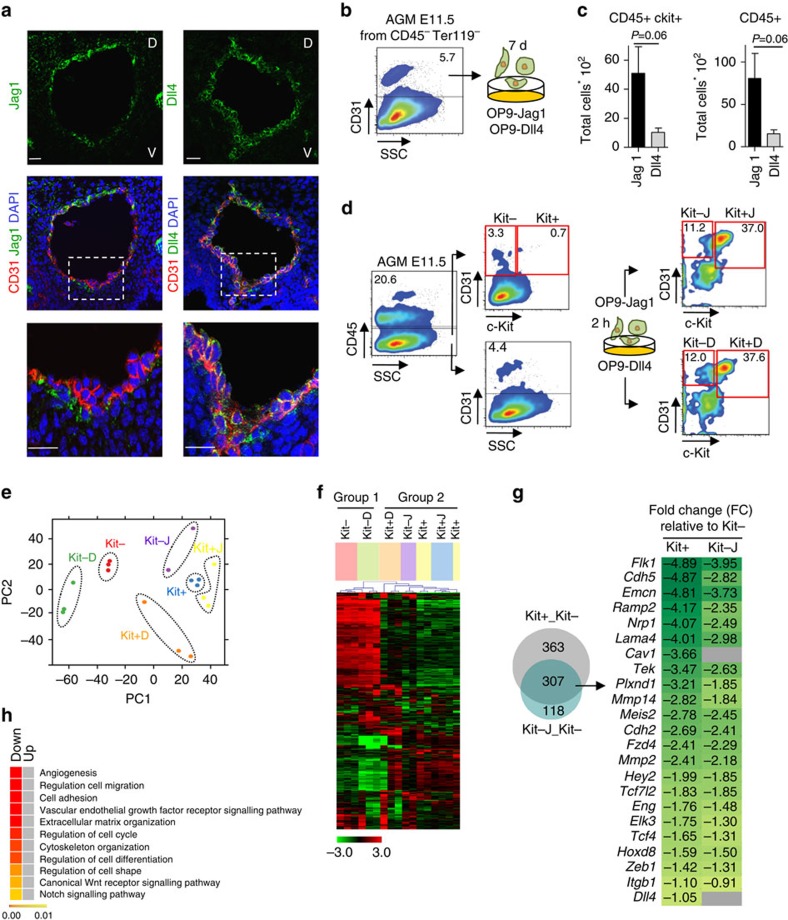
Expression of Jag1 and Dll4 ligands in the embryonic dorsal aorta. (**a**) Representative confocal images of E10.5 embryo transverse section with CD31 (red) and Jag1 (green, left) and Dll4 (green, right). Details of ventral part (lower panels) corresponding to boxed areas. Scale bars, 25 μm. Nuclear staining with 4,6-diamidino-2-phenylindole is shown (D, dorsal; V, ventral). (**b**) Experimental design to test the effects of OP9-Jag1 and OP9-Dll4 on purified CD31^+^CD45^−^Ter119^−^ AGM cells after 7 days of culture. (**c**) Quantification of haematopoietic lineage generated from CD31^+^CD45^−^Ter119^−^ AGM cells on culture on OP9-Jag1 or OP9-Dll4. Bars represent the total number of cells on 7-day culture; *n*=4 or more samples of at least two independent experiments. s.e.m. is represented. *P* value for *t*-test is indicated. (**d**) Schematic representation for purification of E11.5 AGM CD31^+^Kit^−^CD45^−^Ter119^−^ and CD31^+^Kit^+^CD45^−^Ter119^−^ (Kit− and Kit+). In parallel, CD31^+^CD45^−^Ter119^−^ cells were incubated for 2 h in OP9-Jag1 or OP9-Dll4. Cells were resorted on the basis of Kit expression (Kit−J and Kit+J or Kit−D and Kit+D). (**e**) Principal component analysis (PCA) of global gene expression profiles of samples included in the study. Each dot of the same colour represents arrays from replicates of the same sample. Dotted lines arbitrarily reunite replicates of a specific condition. (**f**) Unsupervised hierarchy clustering of transcriptional profiles from selected cell populations. (**g**) Venn diagram displaying the number of genes differentially expressed in cluster-containing versus endothelial populations (Kit+_Kit−) and/or in endothelial population after versus before incubation on OP9-Jag1 (Kit−J_Kit−). Fold change expression levels from MicroArray analysis of 23 angiogenic-related genes are listed. Green-colour grade represents the range of fold change (FC) values on Kit+ or Kit−J populations compared with Kit− cells. All FC values represent statistically significant differences on gene expression (*P* value-adjusted<0.05). Grey cells represent genes in which FC is not statistically significant. (**h**) Biological process enrichment analysis of genes differentially expressed in Kit−J_Kit− and Kit+_Kit− comparisons. Only selected GO terms are presented, and all significant terms are given in Supplementary Tables 2 and 3. (**e**–**h**) Analysis performed on three independent experiments; one Kit−J sample was excluded for technical reasons.

Both Dll4 and Jag1 ligands were strongly and homogeneously distributed along the rostral-caudal and dorsoventral axis of the aortic endothelium by E10.5 (Fig. 2a and Supplementary Fig. 2a). Interestingly, Kit^+^ cluster structures showed variable patterns of ligand distribution, most frequently being the positive signal for Dll4 (Supplementary Fig. 2b). However, the majority of sorted Kit^−^ endothelial cells (89%) co-expressed the Jag1 and Dll4 ligands, and only a few cells expressed either Jag1 (3.8%) or Dll4 (4.6%) or were negative for both (2.5%; Supplementary Fig. 2d). At the transcriptional level, lower levels of ligand mRNA were detected in the Kit+ population (cluster cells) compared with the endothelial (Kit−) population (Supplementary Fig. 2e).

### Jag1 inhibits the endothelial programme in AGM cells

The ligand distribution observed in the aortic endothelium of the AGM suggests the existence of a functional interplay between Jag1 and Dll4. Thus, we aimed to investigate the relative contribution of each ligand in the process of haematopoietic and endothelial production from AGM endothelial progenitor cells. With this purpose, we dissected E10.5 and E11.5 AGM tissues and sorted the cells based on CD31 positivity (excluding Ter119^+^ and CD45^+^ cells). We then incubated the CD31+ AGM cells for 7 days in the presence of OP9 stroma overexpressing either Jag1 (OP9-Jag1) or Dll4 (OP9-Dll4) ligands (Supplementary Fig. 3c) and analysed the cultures for the presence of cells expressing the haematopoietic markers Kit and CD45, as a measure of haematopoietic cell production. Generation of haematopoietic cells (CD45^+^) and haematopoietic progenitors (Kit^+^CD45^+^) from E10.5 and E11.5 AGM cells was increased both in percentage (eightfold increase; Supplementary Fig. 3d) and in total cell number (Fig. 2c) when comparing OP9-Jag1 with OP9-Dll4 cultures. To investigate whether Jag1 and Dll4 affect the haematopoietic cell production by regulating the EHT process, we performed a similar experiment; however, in this case the sorted CD31^+^CD45^−^ E11.5 AGM cells were incubated on OP9-Jag1 or OP9-Dll4 for 2 h, resorted according to Kit expression and processed for transcriptome analysis using Affymetrix arrays. Of note that the final analysis included four populations generated in the experiment (Kit− and Kit+ after incubation with Jag1 (Kit−J and Kit+J) or with Dll4 (Kit−D and Kit+D)) and two controls corresponding to the untreated populations (CD31^+^Kit^−^CD45^−^ (Kit−) and CD31^+^Kit^+^CD45^−^ (Kit+)) that were directly sorted from freshly dissected E11.5 AGMs (Fig. 2d). Independent triplicates of each sample were obtained and processed using the Affymetrix gene chips. Bioinformatic analysis detected global changes in gene expression on ligand incubation, relative to the basal transcriptome of the Kit+ and Kit− cell populations. Principal component analysis (Fig. 2e) detected a high degree of similarity between replicates and a clear intrasample segregation of the replicate clusters on incubation with the ligands (Kit−J, Kit+J, Kit−D and Kit+D) from untreated Kit+ and Kit− AGM cells. Two-dimensional representation of principal component space shows a recognizable distribution, in which Kit− and Kit−D samples segregate not only from all Kit+ samples (Kit+, Kit+J and Kit+D) but also from the Kit−J. Similarly, unsupervised hierarchical clustering algorithm identified the following two transcriptional groups: (1) the untreated endothelial cells (Kit−) clustered with endothelial cells exposed to Dll4 (Kit−D) and (2) the haematopoietic precursor populations (Kit+, Kit+D and Kit+J) that clustered together with the Kit− cells exposed to Jag1 (Kit−J; Fig. 2f). Together, these analyses strongly suggest that Jag1 imposes a haematopoietic-like signature to the Kit− population, before the acquisition of detectable amounts of the Kit haematopoietic marker.

Next, we compared the expression profiles of the endothelial population exposed to Jag1 (Kit−J), the haematopoietic precursors (Kit+) and the endothelial population (Kit−). Unexpectedly, we did not identify a shared haematopoietic signature in Kit+ and Kit− cells exposed to Jag1 (Kit−J). Instead, exposure of the Kit− population to Jag1 was associated with downregulation of several endothelial-related genes on the Kit− population such as *Flk1*, *Nrp1* and *Cdh2* among others (Fig. 2g and GSE59344). Accordingly, Gene Ontology (GO) analysis of the data indicated that genes downregulated in the Kit− cells by exposure to Jag1 fall into categories related to specification and maintenance of endothelial cell identity (including angiogenesis, regulation of cell migration and cell adhesion), or are associated with the Notch and Wnt pathways (Fig. 2h and Supplementary Tables 2 and 3).

**Figure 3:**
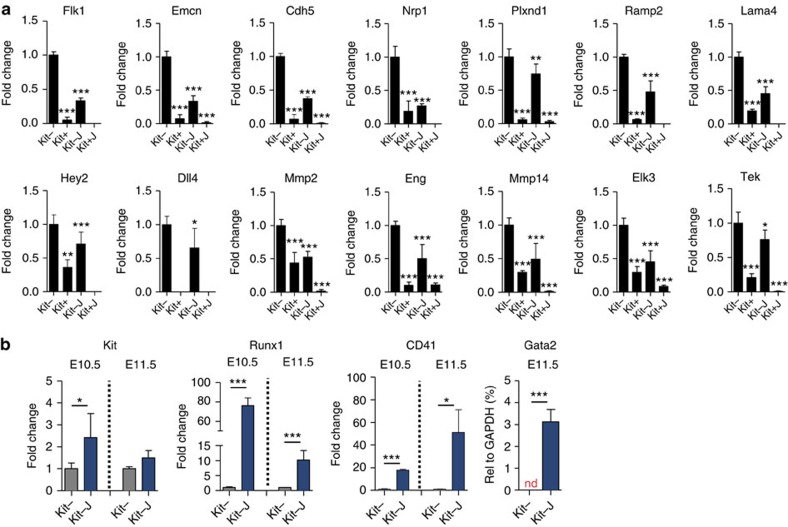
Jag1 induces the downregulation of the endothelial signature. (**a**) Microarray validation using qRT–PCR. Expression of a panel of endothelial-related genes on E11.5 CD31+CD45−Ter119− AGM cells incubated 2 h on OP9-Jag1. Quantification was performed in Kit−, Kit+, Kit−J and Kit+J. The bars represent the average expression level of four to six replicates from two independent experiments, normalized to Kit− expression. Average fold change (±s.d.) is represented. Student's *t*-test for comparisons of Kit+, Kit−J and Kit+J populations with Kit− population was performed (*P≤0.05; **P≤0.01; *P*≤0.001). (**b**) Expression of haematopoietic genes on E10.5 and E11.5 Kit− and Kit−J populations (2 h on OP9-Jag1). The bars represent the average (±s.e.m.) expression level of six to nine replicates from three or four independent experiments, normalized to Kit− expression. Statistical significance was assessed by Student's *t*-test (*P≤0.05; ***P≤0.001 when significant). nd, not detected.

To confirm that Jag1 imposes the downregulation of a pre-existing endothelial signature in the Kfit− cells, we performed reverse transcriptase–PCR (RT–PCR) analysis of genes identified in our GO analysis (*n*=39) from independent pools of cells sorted and processed as detailed above. The 95% of all tested genes were found to be downregulated in the different pools of Kit+ cells when compared with Kit− AGM cells, as expected. Importantly, in two independent experiments 62% of all tested genes showed a reduction in their mRNA levels in the Kit−J compared with the original endothelial population (Kit−; Fig. 3a). Comparable results were obtained from sorted endothelial CD31^+^Kit^−^CD45^−^ cells grown on the OP9-Jag1 for 2 or 5 h (Supplementary Fig. 4a,b, respectively). These results exclude the possibility that the observed transcriptional changes are originated from Kit+ cells that lost the Kit marker during Jag1 incubation. In addition, we detected a general decrease in flk1 levels in the entire endothelial population after 5-h incubation on Jag1 as determined using flow cytometry (Supplementary Fig. 4c), suggesting that the effects of ectopic Jag1 are not restricted to a minor haemogenic endothelial cell population.

Altogether, the results strongly suggest that Jag1 counteracts a transcriptional endothelial programme that is likely imposed by Dll4 in the Kit− cell population.

### Jag1 enhanced haematopoietic gene expression

The EHT process involves the acquisition of a haematopoietic transcriptional programme that was not detected as differentially regulated in our microarray analysis. Thus, we specifically determined the expression levels of particular haematopoietic genes in the Kit− and Kit+ AGM cell populations, and in comparison with the Jag1-incubated Kit− cells (Fig. 3b). We detected a remarkable upregulation of *Kit*, *CD41*, *Runx1* and *Gata2* expression in E10.5 or E11.5 Kit− AGM cells exposed to Jag1 (Fig. 3b and Supplementary Fig. 4b), expected from cells undergoing haematopoietic commitment. Taken together, our results suggest that Jag1 promotes the EHT process through both the downregulation of the endothelial signature and the activation of the haematopoietic-specific transcription.

### Jag1 is required to inhibit the endothelial signature in EHT

**Figure 4:**
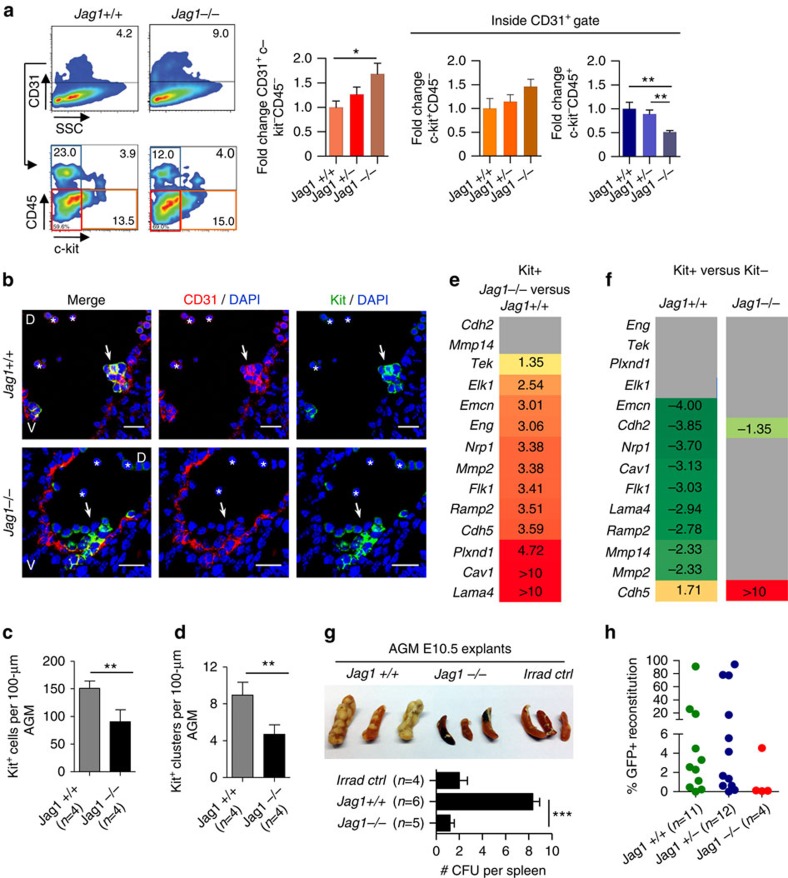
Jag1 mutants fail to generate functional HSCs and retain endothelial signature. (**a**) Representative analysis of population distribution in *Jag1*+/+, *Jag1*+/− and *Jag1*−/− animals. Endothelial population (CD31^+^Kit^−^CD45^−^) in red (frame and graph bars), cluster-containing population (CD31^+^Kit^+^CD45^−^) in orange (frame and graph bars) and mature haematopoietic population (CD31^+^Kit^−^CD45^+^) in blue (frame and graph bars). Bars represent the average population distribution in *Jag1*+/+ (*n*=6), *Jag1*+/− (*n*=10) and *Jag1*−/− (*n*=6) embryos, normalized to *Jag1*+/+ ±s.e.m. Significance was assessed by Student's *t*-test (*P≤0.05 and **P≤0.01 when significant). (**b**) Representative confocal images of transversal sections of E10.5 AGM from *Jag1*+/+ and *Jag1*−/− stained for CD31 (red) and Kit (green). Nuclear staining with DAPI. White arrows point to cluster-like structures. Scale bar, 25 μm. Asterisks indicate autofluorescent circulating cells. D, dorsal; V, ventral. (**c**,**d**) Quantification of Kit+ cells (**c**) and Kit+ clusters (group of at least four positive cells; **d**) per 100 μm of AGM from *Jag1*+/+ and *Jag1*−/− embryos. Bars represent the average±s.e.m. of cells per embryo (*n*=4). Student's *t*-test was performed (**P≤0.01). (**e**,**f**) FC in the expression levels of the indicated genes as detected using qRT–PCR from E10.5 Jag1−/− compared with Jag1+/+ Kit+ cells (**e**) or in Kit+ compared with Kit− populations in the Jag1+/+ and Jag1−/−. (**f**). Two independent pooled samples for each genotype were analysed. Colour grades reflect the FC values of each gene expression. All FC values represent statistically significant differences on gene expression (*P* value≤0.05). Genes that do not show a statistically significant alteration in pairwise comparison are listed in grey. See also Supplementary Fig. 5a. (**g**) CFU-S_11_ from E10.5 *Jag1*+/+ or *Jag1*−/− AGM explant cultures. *n* represents the number of treated animals per condition. Each mouse was transplanted with one embryo equivalent AGMs on 3 days in explant culture. Results obtained from three independent experiments. Irrad ctrl: irradiated/non-injected control. Bars represent the mean±s.e.m. of colonies per tissue. Significance is assessed by Student's *t*-test (***P≤0.001; not assigned if not significant). (**h**) Percentage of reconstitution at 4 months post transplantation. Each dot represents a single animal. *n* represents the number of transplanted mice per genotype.

*Jag1*−/− embryos die around E11 with impaired definitive haematopoietic development^7^. A detailed analysis of the subpopulations present in the *Jag1*−/− AGM at E10.5 identified an increase in the number of endothelial-like cells (CD31^+^Kit^−^CD45^−^), but also CD31^+^Kit^+^CD45^−^ haematopoietic precursors, associated with a decline in the number of CD45+ cells (Fig. 4a). Immunofluorescence analysis of AGM sections confirmed the presence of Kit+ cells in the *Jag1*−/− embryos; however, both the total number and the morphology of these haematopoietic-like clusters were severely affected (Fig. 4b–d). Specifically, Jag1-deficient Kit+ cells were distributed as single cells along the aortic endothelium or aggregate in clusters that do not evaginate into the aortic lumen, thus invading the mesenchymal tissue underneath the endothelial layer instead (Fig. 4b). To characterize the haematopoietic defects in Jag1−/− embryos at the molecular level, we sorted E10-5 AGM CD31^+^Kit^−^CD45^−^ (Kit−) and CD31^+^Kit^+^CD45^−^ (Kit+) cells from *Jag1+/+* and Jag1−/− embryos, obtained the total RNA from each population and compared their gene transcriptional patterns using qRT–PCR. Our results showed that *Jag1*−/− embryos have a prominent upregulation of the endothelial-related signature both in the Kit+ and Kit− populations compared with *Jag1*+/+ (Fig. 4e, Supplementary Fig. 5a). More importantly, comparison of Kit+ and Kit− populations demonstrated that *Jag1*−/− cells maintain a consistent endothelial signature during the Kit− to Kit+ transition (Fig. 4f). Further evidence for this endothelialization of the Kit+ cell population in the *Jag1*−/− embryos is provided by the observed decrease in the expression of C-Kit and *Runx1* when compared with Jag1+/+ littermates (Supplementary Fig. 5b).

Because HSCs are known to reside inside the Kit^+^CD45^+^ population, which is still present in the *Jag1*−/− embryos (not shown), we functionally measured the frequency of haematopoietic progenitors (CFUs-S_11_) and HSCs in these mutant embryos compared with *Jag1+/+* littermates. We established E10 AGM explant cultures from *Jag1*+/+, *Jag1*+/− and *Jag*1−/− embryos and, after 72 h, cells were injected into lethally irradiated mice. On day 11 post transplantation, we found that *Jag1*−/− AGM-injected mice contained a very low number of colonies in the spleen (colony-forming units in spleen, CFU-S_11_) that was comparable to the non-injected/irradiated controls. The number of these colonies was increased up to eightfold in the animals injected with wild-type AGM explant cells (Fig. 4g). To determine the HSC activity, β-actin-GFP;*Jag1*+/+, β-actin-GFP;*Jag1*+/− or β-actin-GFP;*Jag*1−/− AGM explants (from E10 to 10.5 embryos) and transplanted (together with 5 × 10^5^ spleen supporting cells) into lethally irradiated recipients. Donor engraftment was analysed by the presence of GFP at 1 and 4 months after transplantation. We found that 14 out of 23 *Jag1* +/+ or +/− embryos contained long-term multilineage reconstitution activity (engraftment >1% GFP+ cells), whereas we did not detect any HSC activity in the animals transplanted with the *Jag1*−/− AGMs (Fig. 4h).

### Jag1 outcompetes Dll4 signals between AGM cells

**Figure 5:**
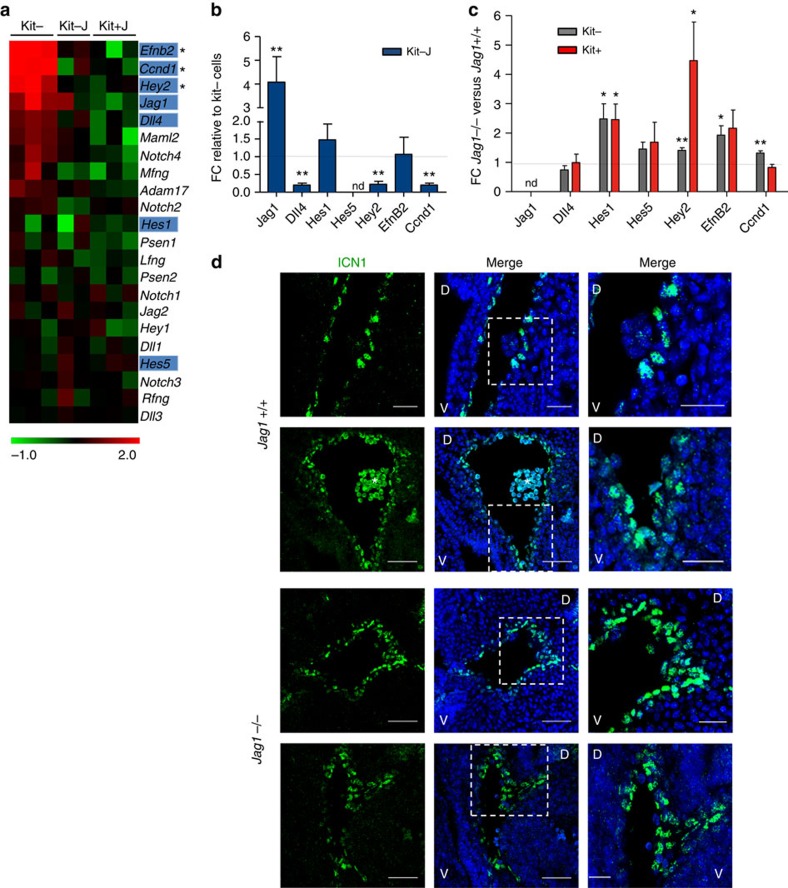
Jag1 attenuates Notch signalling imposed by Dll4 in the AGM. (**a**) Heatmap displaying Notch pathway elements' signature enrichment in E11.5 endothelial population (Kit−) and in Kit− and Kit+ cells on Jag1 incubation of the endothelial fraction (Kit−J and Kit+J, respectively). Highlighted genes are differentially expressed in Kit+_Kit− (Fig. 2); *represents differentially expressed in Kit−J_Kit−. Non-marked genes are not significant (*P*≤0.05). (**b**) Fold change expression levels using qRT–PCR of Notch pathway elements in E11.5 endothelial population on OP9-Jag1 incubation (Kit−J) compared with Kit− cells. The bars represent the average expression of 7 to 12 replicates from at least three independent experiments±s.e.m. Student's *t*-test was performed (**P≤0.01; ***P≤0.001; ns, not significant; n.d., not detected). (**c**) Fold change expression levels using qRT–PCR of Notch pathway elements in Kit− (grey) or in Kit+ population (red) from E10.5 *Jag1*−/− compared with *Jag1*+/+. Bars represent the average expression level of five to six replicates from two independent experiments. Statistical significance was assessed by Student's *t*-test (*P≤0.05; **P≤0.01; not significant, not assigned). (**d**) Confocal images of transverse sections of the dorsal aorta in two E10.5 embryos from *Jag1*+/+ and *Jag1*−/− stained with anti-cleaved Notch1 (green). Detail of the dorsal aorta corresponding to the boxed areas (right panels). *represents autofluorescent circulating cells. Scale bars, 50 μm in *Jag1*+/+, 25 μm in *Jag1*−/− embryos and for magnified regions.

**Figure 6:**
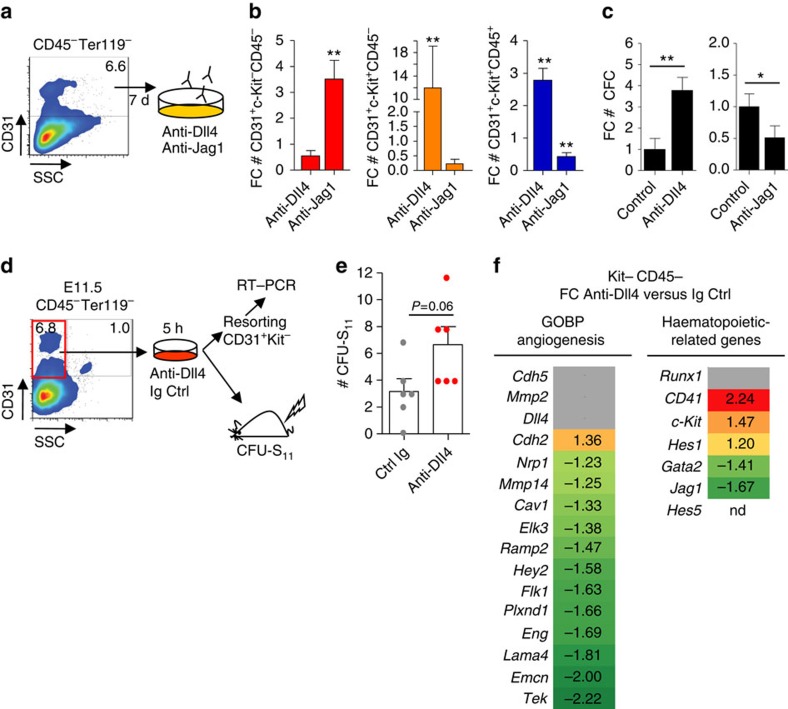
Blockage of Dll4 promotes haematopoietic commitment. (**a**) Schematic of experimental design. E10.5 AGM CD31^+^CD45^−^Ter119^−^ cells were sorted and incubated in the medium with anti-Dll4- or anti-Jag1-blocking antibodies or irrelevant Ig for 7 days. (**b**) Graphs represent the average FC of total cell number obtained after culturing with anti-Dll4 or anti-Jag1 compared with each Ig control±s.e.m. from three independent experiments. Student's *t*-test was used to assess the significance (**P≤0.01; not assigned if not significant). (**c**) Relative number of CFC haematopoietic progenitors obtained in **b**. Average results from three independent experiments ±s.d. determination. Student's *t*-test was used to assess the significance (*P≤0.05; **P≤0.01). (**d**) E11.5 CD31^+^Kit^−^CD45^−^Ter119^−^ cells were sorted and incubated for 5 h on the medium supplemented with anti-Dll4-blocking antibody or irrelevant Ig control. Cells were injected in 3-Gy-irradiated SCID-Beige mice or resorted for mRNA extraction and qRT–PCR analyses. (**e**) Quantification of spleen colonies (CFU-S_11_). Bars represent the average of six replicates from three independent experiments (coloured dots) ±s.e.m. Student's *t*-test was used to assess significance. (**f**) FC in expression levels of a panel of angiogenic- and haematopoietic-related genes using qRT–PCR. Colour grades reflect the fold change values of each gene expression (normalization to expression levels in control conditions). All coloured FC values represent statistically significant differences on gene expression (*P*≤0.05, using Student's *t*-test). Grey is shown for non-statistical significant differences.

Indicating that Jag1 was important to provide a low Notch signal activity in the embryonic haematopoietic precursors, we found that *Hey2*, *EfnB2* and *Ccnd1* (well-known targets of Notch signalling) were markedly downregulated in the Kit− cells exposed to the Jag1-expressing stroma (Fig. 5a,b), while nonsignificant changes in the transcription of these genes were detected in Kit− cells exposed to Dll4 compared with the original kit− population (*P*>0.05). Because most Kit− cells expressed both Dll4 and Jag1, we speculated that Jag1 presented by OP9 cells produces a low Notch signal while outcompeting Dll4-mediated signalling originated from adjacent Kit− cells. In agreement with this possibility, the expression levels of several Notch-target genes were significantly upregulated in the aortic endothelium of Jag1−/− embryos (Fig. 5c) and we detected high Notch activity in multiple areas around the AGM aorta of the Jag1−/− embryos, as determined using immunofluorescence with the antibody recognizing the active form of Notch (ICN1; Fig. 5d). To further investigate whether Jag1 negatively regulates the endothelial programme by competing with Dll4-mediated Notch1 signalling, we evaluated the effect of anti-Dll4 and anti-Jag1-blocking antibodies on the *in vitro* generation of haematopoietic cells from E10.5 AGMs CD31^+^CD45^−^ progenitors (Fig. 6a). After 7 days of culture, anti-Dll4 treatment significantly increased the number of haematopoietic CD31^+^Kit^+^CD45^−^ precursors and CD31^+^Kit^+^CD45^+^ progenitors relative to the IgG control (Fig. 6b,c). Conversely, blocking Jag1 ligand had an inhibitory effect on the generation of these same cell populations (Fig. 6b,c). We next tested the capacity of *in vitro*-generated cells to form functional haematopoietic precursors in the methylcellulose colony-forming assay. We found that, whereas anti-Dll4 treatment increased the number of colony-forming cells in these cultures, anti-Jag1 treatment significantly reduced their colony-forming activity, relative to the controls (Fig. 6c). We therefore evaluated the effects of a transient block of the Dll4 signal in the Kit− population *in vivo* (CFU-S_11_ assay). We incubated Kit− sorted cells for 5 h in the presence of the anti-Dll4 antibody or irrelevant human Ig (as a control) and injected them to sublethally irradiated SCID-BEIJE mice (Fig. 6d). Analysis of the spleens 11 days after injection revealed that animals transplanted with control-treated Kit− cells contained a number of colonies comparable to the uninjected, irradiated control mice. In contrast, we consistently found an increase in the number of spleen colonies in the animals injected with Kit− cells incubated with the anti-Dll4 antibody (Fig. 6e). Moreover, we detected a significant downregulation of the endothelial programme in the Kit− cells incubated with anti-Dll4 for 5 h when compared with the control Kit− cells (Fig. 6f; analogous to the effect observed in the AGM cells incubated on OP9-Jag1, see Fig. 2e–g). These results are consistent with the hypothesis that Jag1 outcompetes Dll4 for binding to the receptor but elicits a weaker activation of Notch1 in the endothelial cells of the AGM.

Altogether, our results indicate that Dll4-induced Notch1 activity is required to specify the arterial programme, while Jag1-induced Notch activity and the subsequent (or concomitant) downregulation of the Dll4-mediated signalling are both required for generating HSC in the embryo. The N1IP::Cre mice data indicate that endothelial and haematopoietic specification associates with a differential Notch signal strength, and we propose that regulation of the correct Notch1 signal strength during embryonic development is achieved by the competition between Dll4 and Jag1 ligands.

## Discussion

Understanding the complex regulatory signals that govern the generation of HSC is clinically relevant for regenerative medicine applications.

In the present study we particularly focused on the regulation and contribution of the Notch pathway during HSC specification in the mouse embryo. We had previously demonstrated that Notch1 activation through the Jag1 ligand was required for the expression of the haematopoietic programme^21^. However, analysis of the Notch1 reporter N1IP::Cre^LO^ (ref. 17) indicated that the haematopoietic lineage was not labelled, and therefore we deduced that the aortic endothelial cells receiving high levels of Notch signal did not produce HSC. Our newly engineered N1IP::Cre^HI^ reporter labelled cells in both the arterial and haematopoietic systems, suggesting that in the dorsal aorta endothelial cells with low Notch1 activity (labelled in the N1IP::Cre^HI^ but not in the N1IP::Cre^LO^) sort out from the ones with high Notch activity (labelled in both lines) to become haematopoietic precursors. Of note that the N1IP::Cre^HI^ activity (as reported by YFP positivity) is detected earlier in development and more intense in the dorsal aorta, and subsequently only the N1IP::Cre^HI^ displays positive cells in the haematopoietic clusters. Our explanation is that the haemogenic endothelium differs from endothelial cells in the magnitude of Notch activity, which will never reach the levels required to induce the N1IP::Cre^LO^ reporter. Alternatively, there is a common precursor that is specified to the arterial or haemogenic lineage during the period in between N1IP::Cre^HI^ and N1IP::Cre^LO^ induction (as detected by YFP).

Analysis of N1IP::Cre^HI^ HSC indicated that YFP+ cells contain bona fide HSCs in fetal liver and bone marrow; however, some long-term repopulating activity is also found in the YFP− population that may have escaped recombination. However, YFP− cells do not show secondary engraftment, strongly suggesting that it correspond to a qualitatively different type of HSCs.

It has been shown that Notch ligands can deliver different Notch signal strengths^20^,^22^. Jag1 and Dll4 ligands are expressed in the embryonic aorta and both can activate the Notch1 receptor. Deletion of either ligand results in distinct endothelial/haematopoietic phenotypes, highlighting a functional difference between the ligands: Dll4 deletion results in an arterialization defect^10^,^12^,^13^, whereas Jag1 deletion specifically affects the establishment of definitive haematopoiesis (ref. 7 and this work).

We combine all available data with our analysis to propose how the balance between Notch1-Dll4 and Notch1-Jag1 signalling guarantees the correct establishment of the endothelial and haematopoietic cell fates in the AGM. Our data indicate that the lack of Jag1 ligand results in higher Notch activity in the aortic endothelium of the AGM, which enhanced endothelial fates at the expense of HSC formation. This observation supports existing models in which Dll4-Notch1 signals maintain the endothelial/arterial programme. We hypothesize that precursor haemogenic cells responding to Jag1 attenuate/inhibit the strong Dll4-Notch1 signal, replacing it with a productive low Notch1 signal necessary and sufficient to activate haematopoietic genes, such as *Gata2* (ref. 21), but not to activate the endothelial programme. In addition, we found that Jag1 induced the transcriptional activation of several microRNAs (GSE59344) that may contribute to the active repression of the endothelial programme, a possibility that we are currently investigating. The requirement of a Jag1-dependent productive Notch signal for haematopoietic specification is further supported by the fact that treatment of AGM cells with anti-Jag1-blocking antibody prevents production of haematopoietic progeny similar to γ-secretase inhibition^7^. Finally, although our results obtained in the OP9-Jag1 co-culture experiments are compatible with Jag1 delivering a proliferation signal on the kit+ population, the fact that culture conditions are optimized for haematopoietic cell growth precluded to obtain any conclusion about target-cell specificity. Moreover, these effects of Jag1 on cell proliferation are not supported by gene expression analysis on the kit+J population.

Antagonistic interactions between Jagged and Delta ligands to attenuate Notch signalling have been previously proposed for angiogenic sprouting^23^ or prosensory specification in the inner ear^22^. In the vascular network of the retina, Jag1 antagonizes strong Dll4-Notch1 signalling in a stalk cell to promote the tip fate that requires a weak/no Notch1 signal. Fringe enhances the ability of Notch1 to respond to Dll4 while lowering activation by Jag1 in the tip and the developing inner ear^22^,^24^. Fringe is likely to be involved in EHT; however, the specific role of fringe in HSC development has not been addressed yet.

Lines of evidence in different organisms support the model known as *Cis*-inhibition, in which activation of Notch by a ligand expressed in a neighbouring cell (sending cell) is prevented by the ligands that are co-expressed with Notch in the receiving cell^25^,^26^,^27^,^28^,^29^. Our observation that endothelial genes in Kit− cells are repressed by incubation with OP9-Jag1 (signal in *trans*) points against a pure *cis*-inhibitory model, thus suggesting the existence of a *trans*-inhibition mechanism.

The importance of Jag1 signal in the maintenance of the haematopoietic identity was previously demonstrated in the *Jag1*−/− mice^7^; however, a precise analysis of the haemogenic and HSC subpopulations in these animals was lacking. We now show that Kit+ cells are produced in Jag1-deficient aortas in a similar frequency as the wild type; however, mutant cells fail to downregulate the endothelial programme, are not properly localized in the emerging clusters and show a subaortic mesenchymal localization.

In summary, our study identifies a novel two-step process in the specification of definitive HSC by Jag1 during mammalian embryonic development. First, Jag1 must protect the progenitors from Dll4 signals, which leads to downregulation of the endothelial signature. Second, Jag1 signals are required for endothelial-to-haematopoietic transition in the haemogenic endothelium, and for the acquisition of the haematopoietic phenotype. This knowledge has great importance for the future design of protocols for *in vitro* generation of HSCs, which is a relevant issue in regenerative medicine, in particular for producing cells suitable for transplantation in patients without compatible blood donors.

## Methods

### Animals

CD1, C57BL/6 J wild-type, B6.SJL-Ptprca Pep3b/BoyJ, SCID-Beige mice (Charles River Laboratories), Jag1−/− (ref. 30), β-actin-GFP (ref. 31) and ROSA^eYFP^ (ref. 32) N1IP::Cre^LO^ (ref. 17) and N1IP::Cre^HI^ (ref. 19) strains were used. Animals were kept under pathogen-free conditions, and all procedures were approved by the Animal Care Committee of the Parc de Recerca Biomedica de Barcelona (regulation of Generalitat de Catalunya). Embryos were obtained from timed pregnant females and staged by somite counting: E10.5 (31–40 sp) E11.5 (43–48 sp). The detection of the vaginal plug was designated as day 0.5. Mice and embryos were genotyped using PCR when justified.

### OP9 cell culture and stromal-free culture of AGM-derived sorted cells

OP9 stromal cell lines overexpressing Jag1 or Dll4 ligands^33^ were maintained in α-minimum essential medium (Gibco, Life Technologies) supplemented with 20% fetal bovine serum (FBS) and 1% penicillin/streptomycin and were incubated at 37 °C in a humidified atmosphere with 5% CO_2_. Cells were plated at 1.5 × 10^4^ cells cm^−2^ 24 h before experiment.

Sorted cells were plated in Iscoves medium (Gibco, Life Technologies) supplemented with 10% inactivated FBS, 10 ng ml^−1^ interleukin (IL)-3, 10 ng ml^−1^ stem cell factor (SCF), 20 ng ml^−1^ IL-6, 10 ng ml^−1^ insulin-like growth factor-1 (IGF-1), 10 ng ml^−1^ fibroblast growth factor-basic (FGF-B), 10 ng ml^−1^ vascular endothelial growth factor (VEGF), 2 U ml^−1^ erythropoietin, 4.5 × 10^−4^ M monothioglycerol, 10 μg ml^−1^ Heparin and 50 ng ml^−1^ bovine pituitary extract.

Cells were incubated for 2, 5 h or 7 days depending on experiment. Incubation with 1 μg ml^−1^ of blocking anti-Jag1 N-17 (sc-34473, Santa Cruz Biotechnology), 5 μg ml^−1^ of blocking anti-Dll4 (Genentech) or with the Ig mock controls at the correspondent concentration (irrelevant Goat IgG, Sigma I9140 or Goat Anti-Human Ig, Southern Biotech 2010-01, respectively).

### Haematopoietic progenitor assay

On culture, AGM-derived cells were harvested and seeded in duplicates in Methocult M-3434 semi-solid medium (Stem Cell Technologies). Cells were incubated at 37 °C with 5% CO_2_ and colony-forming units were counted after 5 days.

### CFU-S_11_

AGM-derived cells on culture were harvested and washed with PBS. Cells were resuspended in 330 μl per sample and injected intravenously into adult sublethally irradiated (3 Gy) C57BL/6J wild-type or SCID-Beige recipients (Figs 4 and 6, respectively). After 11 days, the animals were killed and the presence of macroscopic haematopoietic colonies in the spleen was scored under a stereoscope (KL200 LED; Leica).

### Single-cell suspensions and antibody staining

AGMs were dissected from embryos at E10.5 or E11.5, incubated for 20 min at 37 °C in 0.12% collagenase (Sigma-Aldrich) in PBS+10% FCS and dissociated by pipetting to single-cell suspensions. Cultured cells or primary cells were washed with PBS+10% FBS before antibody staining. Antibody staining was performed in PBS supplemented with 10% FCS in the dark, at room temperature for 15 min, or carried out on ice for 30 min. The antibodies CD45-PeCy7, CD45-FITC, Ter119-PeCy7, CD31-PE and Kit-APC were purchased from BD Biosciences. Dead cells were excluded using Hoechst 33258 (Invitrogen) or 4,6-diamidino-2-phenylindole (DAPI; Invitrogen).

### Flow cytometry and cell sorting

Flow cytometry analysis was performed on FACSCalibur (BD Biosciences) or LSRII (BD Biosciences). Cell sorting was performed on FACSVantage (70-μm nozzle), FACSAria (85-μm nozzle) or Influx (100-μm nozzle; all BD Biosciences). The data were analysed with the FlowJo software (Tree Star) or FACSDiva software (BD Biosciences). Sorted cells were collected either in medium or Qiagen RLT buffer (for culture or mRNA extraction, respectively). When possible, cells were analysed for sorting purity; neither Kit^+^ nor CD45^+^ cell contamination was detected in Kit− population.

### cDNA amplification and quantitative RT–PCR

Total RNA was extracted using the RNeasy Mini Kit (Qiagen). cDNA was obtained with RT First Strand cDNA Synthesis (GE Healthcare) according to the manufacturer's instructions. cDNA was pre-amplified before qRT–PCR reaction using TaqMan PreAmp Master Mix Kit (Applied Biosystems) according to the manufacturer's instructions. The primers used for pre-amplification and qRT–PCR are listed in Supplementary Table 1.

### Microarray analysis

For microarray study, AGM regions were obtained from E11.5 mouse embryos, digested with 0.1% collagenase and single-cell suspension stained with anti-Ter119, anti-CD45 and anti-CD31. The CD31^+^CD45^−^Ter119^−^ population was sorted in Iscoves-based medium and seeded on OP9-Jag1 or OP9-Dll4 stromal cells for 2 h, as described above. After 2 h, cells from each of the culture conditions were re-stained with anti-CD45, anti-CD31 and anti-Kit, and CD31^+^Kit^−^CD45^−^ and CD31^+^Kit^+^CD45^−^ directly recovered in RLT buffer (Qiagen). Total RNA from three independent sorting experiments was extracted using RNeasy midi or mini Kit (Qiagen) and was assessed using Bioanalyzer 2100 (Agilent Technologies, Palo Alto, CA). Only samples with high integrity (RNA integrity number>7) were subsequently used in microarray experiments. Microarray expression profiles were obtained using the Affymetrix GeneChip Mouse Gene 1.0 ST array (Affymetrix, Santa Clara, CA) and the GCS3000 Affymetrix platform. Briefly, 4.8 ng of total RNA from each sample was amplified using the Ovation Pico WTA System (NuGEN Technologies, San Carlos, CA) and sense transcript cDNA (ST-cDNA) was generated using the WT-Ovation Exon Module (NuGEN Technologies). After, ST-cDNA was fragmented and labelled with the FL-Ovation cDNA Biotin Module V2 (NuGEN Technologies), and the biotinylated cDNA was hybridized to Affymetrix GeneChip Mouse Gene 1.0 ST arrays. Following hybridization, the array was washed and stained, and finally scanned to generate CEL files for each array. Quality metrics on microarray data sets was performed with QualityMetrics 3.14.0 under R version 2.15.2 (2012-10-26). Data have been submitted to GEO (GSE59344; link for reviewers: http://www.ncbi.nlm.nih.gov/geo/query/acc.cgi?token=uhoneeaifnczpmn&acc=GSE59344). Microarray profiles of endothelial population CD31^+^Kit^−^CD45^−^Ter119^−^ and cluster-containing population CD31^+^Kit^+^CD45^−^Ter119^−^ directly sorted from freshly treated AGMSs were obtained elsewhere^34^ and data previously submitted as GSE35395.

### Computational analysis

Hierarchical clustering was performed using Euclidean distance algorithm on median-centred expression value of the genes set that pass the filter of s.d.⩾1. Clustering, heatmap generation and calculation were performed using the programme Genesis^35^. Principle component analysis was performed using Bioconductor^36^ package arrayQualityMetrics^37^.

### Immunostaining

For tissue-section immunostainings, embryos were fixed overnight in 4% paraformaldehyde (Sigma-Aldrich) at 4 °C, included in paraffin or Optimal Cutting Temperature (OCT) (Tissue-Tek, Sakura) and sectioned at 8 μm. Primary antigen retrieval of paraffin-embedded embryos was performed in 10 mM sodium citrate pH 6, 20 min in autoclave. On peroxidase exhaustion (3% H_2_O_2_), slides were incubated in blocking solution (3% BSA, 20 mM MgCl_2_, 0.3% Tween20 and 5% FBS in PBS). Primary antibodies were used at the following concentrations: anti-Jag1 1:400 (sc-6011-Santa Cruz), anti-Dll4 1:3000 (ab7280-Abcam), anti-ICN1 1:100 (α-N1Icv monoclonal antibody Cell signaling #4147S) and developed using horseradish peroxidase (HRP)-conjugated specific secondary antibody ((1:200, Dako) or non-diluted HRP-conjugated universal secondary antibody reagent or secondary EnVision+System-HRP Labelled Polymer Anti-Rabbit (Dako) and tyramide amplification system TSA (PerkinElmer).

OCT-embedded embryos were fixed with −20 °C methanol for 15 min and block-permeabilized in 10% FBS, 0.3% Surfact-AmpsX100 (Pierce) and 5% non-fat milk in PBS for 90 min at 4 °C. Anti-Kit 1:50 (BD 553356) and signal developed using HRP-conjugated specific secondary antibody (Dako) and tyramide amplification system TSA (PerkinElmer). On a step of endogenous biotin blocking (Endogenous Avidin+Biotin Blocking System, ab3387 abcam), CD31 detection with biotinylated anti-CD31 1:500 (BD 553371) followed by incubation with secondary streptavidin Alexa Red 555-conjugated antibody 1:500 (BD 32355).

For sorted cell immunostaining, 2,000 endothelial cells (CD31^+^Kit^−^CD45^−^Ter119^−^) from E11.5 embryos were directly sorted on pre-treated slides and fixed for 40 min on ice in 4% paraformaldehyde. Primary antibodies anti-Jag1 1:400 (sc-6011-Santa Cruz) and anti-Dll4 1:3,000 (ab7280-Abcam) incubation and detection was achieved with specific secondary Alexa 1:500 (A-11056 Alexa Fluor 546 or A-21206 Alexa Fluor 488 (Invitrogen Molecular Probes), respectively.

Whole-mount immunostainings were achieved as detailed in ref. 38. Briefly, embryos were fixed for 20 min on ice in 2% paraformaldehyde, dehydrated in methanol and trimmed. On transference into scintillation vials containing 100% methanol, embryos were rehydrated and blocked with BSA/PBS-MT solution on ice for 1 h. Primary antibodies were used at the following concentrations: anti-Kit 1:500 (eBioscience 14-1171-81), anti-Jag1 1:50 (Santa Cruz sc-6011), anti-Dll4 1:500 (Abcam ab7280) and biotinylated anti-CD31 1:500 (BD 553371). Detection was achieved with secondary Alexa 1:500 (A-21206 Alexa Fluor 488, A-21247 Alexa 647, Invitrogen Molecular Probes) or streptavidin Alexa Red 555-conjugated antibody 1:500 (BD 32355). Vectashield medium plus DAPI (Vector) was used for mounting and nuclear staining. All immunostainings were analysed in a fluorescence microscope (Olympus BX61), and images were taken in a confocal microscope (Leica SP5; lasers of 488-, 561- and 633-nm wavelengths). Fiji imaging and Photoshop were used for final image processing.

## Additional information

**Accession codes:** Microarray data have been deposited in the GEO database under accession codes GSE59344 and GSE35395.

**How to cite this article:** Gama-Norton, L. *et al*. Notch signal strength controls cell fate in the haemogenic endothelium. *Nat. Commun.* 6:8510 doi: 10.1038/ncomms9510 (2015).

## Supplementary Material

Supplementary Information Supplementary Figures 1-5 and Supplementary Tables 1-3
