# Corrigendum: Notch signal strength controls cell fate in the haemogenic endothelium

**DOI:** 10.1038/ncomms10978

**Published:** 2016-03-04

**Authors:** Leonor Gama-Norton, Eva Ferrando, Cristina Ruiz-Herguido, Zhenyi Liu, Jordi Guiu, Abul B. M. M. K. Islam, Sung-Uk Lee, Minhong Yan, Cynthia J. Guidos, Nuria López-Bigas, Takahiro Maeda, Lluis Espinosa, Raphael Kopan, Anna Bigas

Nature Communications 6: Article number: 8510 10.1038/ncomms9510 (2015); Published: 10142015; Updated: 03042016

The original version of this Article contained a typographical error in the spelling of the author Zhenyi Liu, which was incorrectly given as Zenhy Liu. This has now been corrected in both the PDF and HTML versions of the Article.

