# Supplementary material for: Notch signal strength controls cell fate in the haemogenic endothelium
Source: Nat Commun. 2015 Oct 14;6:8510. doi: 10.1038/ncomms9510 (PMC4634136; doi:10.1038/ncomms9510)
Supplement: Supplementary Information — Supplementary Figures 1-5 and Supplementary Tables 1-3 [file ncomms9510-s1.pdf]

SUPPLEMENTAL INFORMATION

Supplementary Figure 1

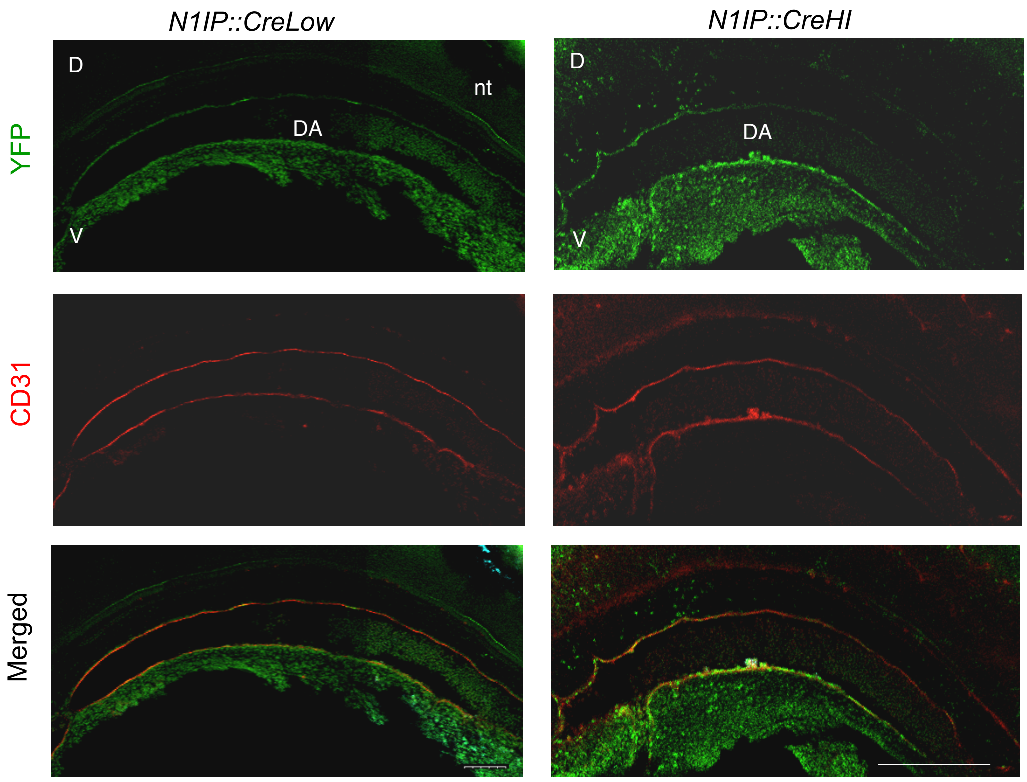

Supplementary Figure 1: **Endothelial YPF labeling of N1IP::Cre^{HI} and N1IP::Cre^{LO}** Representative confocal images of the dorsal aorta from 3D whole-mount staining of E10.5 embryos. Staining for YFP (green) and CD31 (red). D, dorsal; V, ventral; DA, Dorsal Aorta, NT, Neural tube. Scale bars: 100μm for N1IP::Cre^{LO} and 250μm for N1IP::Cre^{HI}.

Supplementary Figure 2

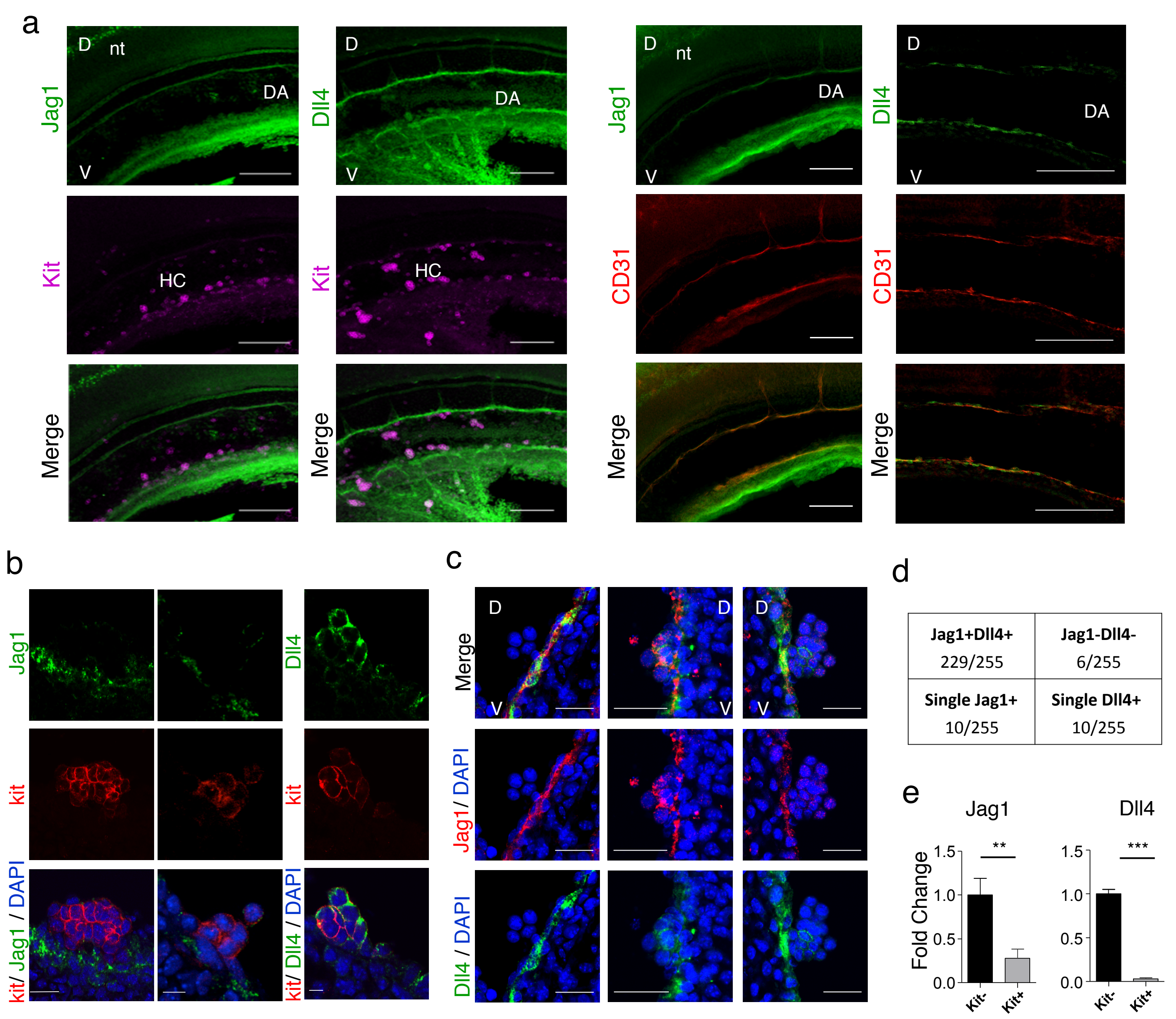

Supplementary Figure 2 : Jag1 and Dll4 distribution on AGM,

a) Representative confocal images of E10.5 embryo. Jag1 or Dll4 (green) and Kit (magenta) in right panels or CD31 (red) in left panels. Scale bar: 100µm. DA, Dorsal Aorta, nt, neural tube; HC, Hematopoietic Cluster.

b) Detail of budding cluster-like structures from E10.5 embryo. Scale bar: 25µm (left panels), 10 µm (middle panels) and 75 µm (right panels).

c) Confocal images of budding cluster from E10.5 AGM embryo displaying different patterns of Jag1 (red) and Dll4 (green). Scale bar: 25µm. Nuclear staining with Dapi is shown in B-C. Dorso-ventral orientation in A (D, dorsal; V, ventral).

d) Cell counts of sorted $CD31^{+}Kit^{-}CD45^{-}Ter119^{-}$ with Jag1 and/or Dll4 expression among 255-screened cells.

e) Relative expression of Jag1 and Dll4 mRNA on endothelial population ("Kit-", $CD31^{+}Kit^{-}CD45^{-}Ter119^{-}$, black bars) and cluster-containing populations ("Kit+", $CD31^{+}Kit^{+}$ $CD45^{-}Ter119^{-}$, gray bars) from E11.5 embryos. The bars represent the average expression level of 12 replicates from 4 independent experiments, normalized to Kit- expression ± SEM. Statistically significance was assessed by Student's *t* test (**p≤0.01; ***p≤0.001).

Supplementary Figure 3

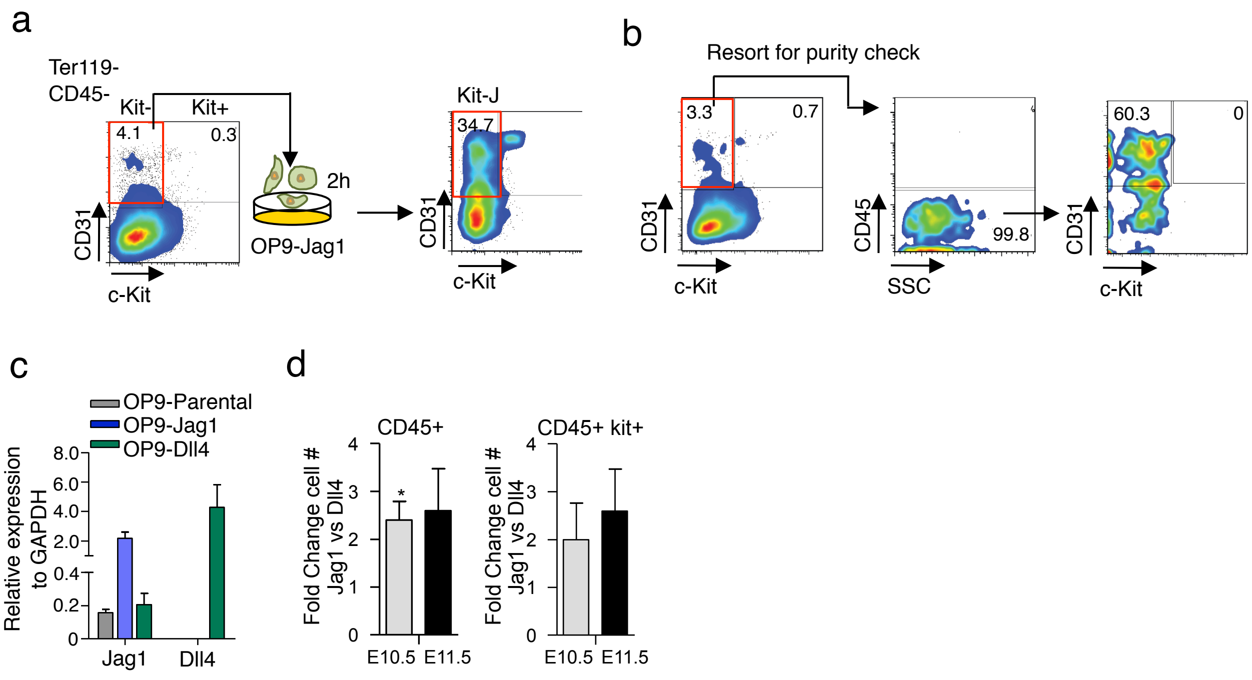

Supplementary Figure 3: kit+ and kit- incubation on OP9-Jag1 and OP9-Dll4

a) Gating strategy used to sort CD31^{+}Kit^{-}CD45^{-}Ter119^{-} endothelial population (Kit-). Upon 2 hours incubation on OP9-Jag1, CD31^{+}CD45^{-}Kit^{-} cells were sorted (Kit-J).

b) Sorting purity associated with the gates used to isolate Kit- cells. The cells were sorted and immediately resorted. No contamination of CD45^{+} cells is observed upon sorting. No Kit expressing cells contaminants are detected in the pool of Kit- sorted cells.

c) Relative expression levels of Jag1 and Dll4 transcripts in OP9-parental, OP9-Jag1 and OP9-Dll4 cell lines by qRT-PCR. Bars represent the mean ±SEM determination from 4 experiments showing the fold change relative to GAPDH.

d) Quantification of CD45^{+} or Kit^{+}CD45^{+} cells “de novo” generated upon culture on stromal cells. Bars represent the relative average fold change (± SEM) in the number of cells generated on OP9-Jag1 stroma compared to OP9-Dll4 culture conditions. For each developmental stage (E10.4 and E11.5), 4 or more samples of at least two independent experiments were considered. Student’s *t*-test was used to assess the significance (**p≤0.01; ***p≤0.001).

Supplementary Figure 4

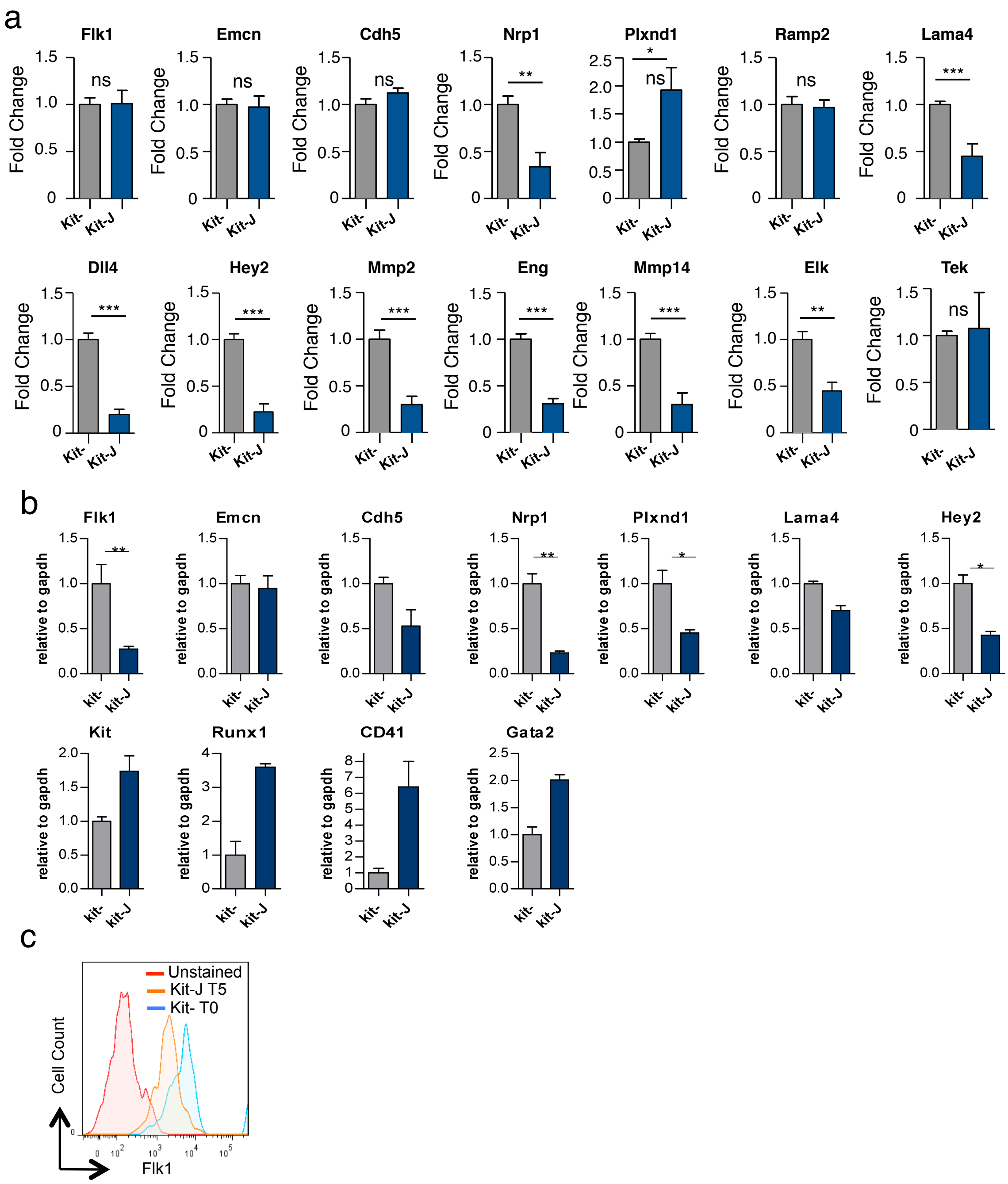

Supplementary Figure 4: Decrease of endothelial and increase of hematopoeitic genes by Jag1.

a) Expression levels of angiogenesis-related genes on E11.5 Kit- and Kit-J populations by qRT-PCR (2h incubation). The bars represent the average expression level of 6 to 9 replicates from 3 independent experiments, normalized to Kit- expression ± SEM. Statistically significance was assessed by Student's *t* test *p≤0.05; **p≤0.01; ***p≤0.001; ns-not statistically significant).

b) Expression levels of genes on E11.5 Kit- and Kit-J populations by qRT-PCR (5h incubation). The bars represent the average expression levels (± SEM) from 3 independent experiments. Statistically significance was assessed by Student's *t* test *p≤0.05; **p≤0.01; not assigned if non-statistically significant.

c) Fluorescence intensity histogram representing the levels of Flk1 staining on sorted endothelial $CD31^{+}Kit^{-}CD45^{-}Ter119^{-}$ cells at time 0 or after 5h incubation on OP9-Jag1.

Supplementary Figure 5

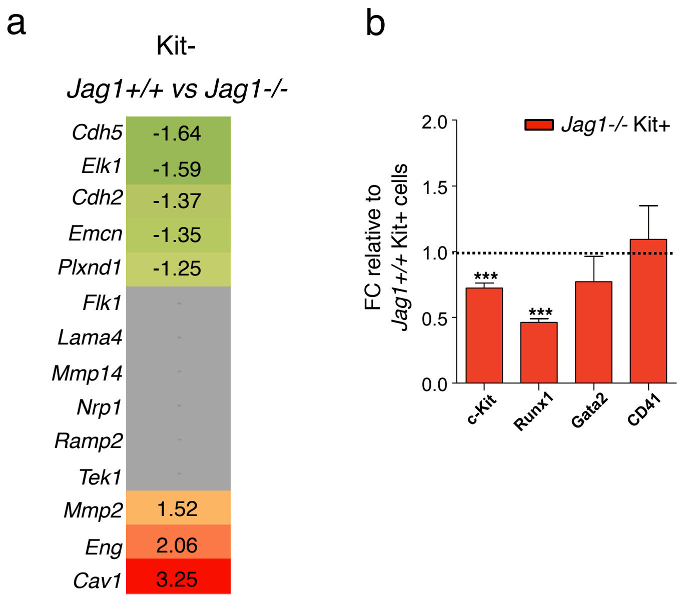

Supplementary Figure 5. Jag1 is required for silencing the endothelial program in Kit+ cells
a) Fold change (FC) expression levels of a panel of angiogenesis-related genes detected in endothelial population (Kit-) of E10.5 *Jag1*+/+ and *Jag*-/- embryos. Cells from each genotype were obtained from 7 sorting experiments were pooled into two independent samples. Color grades reflect the FC values of each gene expression. All FC values represent statistically significant differences on gene expression ($p<0,05$). Genes that do not show a statistically significant alteration in pair-wise comparison are listed in gray.
b) Fold change expression levels by qRT-PCR of hematopoietic-related genes from E10.5 Kit+ fractions from *Jag1*-/- embryos compared to wild-type littermates. The bars represent the average expression level of 4 to 6 replicates from 2 independent experiments ±SEM. Statistically significance was assessed by Student's *t*-test (***$p\leq0.001$; not assigned if not significant).

Supplementary Table 1

Gene Name	Forward Primer	Reverse Primer
Flk1	GGCTGGCATCACCATCAAAAAC	AGGCACAGACACAACAGGGATAGC
Emcn	GCTACCAGGTTCCCAGAACA	ATGCAGAGGATGTTGCAGTG
Cdh2	CATCAATGGCAATCAAGTGG	CATACGTCCCAGGCTTTGAT
Cdh5	TGCATCCTCACCATCACAGT	CAACTGCTCGTGAATCTCCA
Nrp1	GGAACCCAGTGGATGAGTGT	ATGTCGGGAACTCTGATTGG
Plxnd1	GTACCCTGGGCAGAGTGAAA	TACTTTCTTGCGGTGGCTCT
Elk3	AATCTCTGCACCCCAACTGT	AGTCCACTTGGTGTCTGTGC
Mmp14	TTGATGGTGAAGGAGGGTTC	CAGCCACCAAGAAGATGTCA
Ramp2	CTGAGGACAGCCTTGTGTCAA	GTCGCTGTAATGCCTGCTAATC
Lama4	AGCCTCCTGCCTGATGTAGA	GACAAGATGGGTTGCTTGGT
Eng	TTTGTACCCACAAGTCTCGCA	GAGAACGTGGACTTCACGCA
Mmp2	CAGGGCACCTCCTACAACAG	CAGTGGACATAGCGGTCTCG
Hey2	CCTTGTGAGGAAACGACCTC	GTTGTCGGTGAATTGGACCT
Tek	GTAAGCTCAGGTGCCACTGT	GGCCTGCCTTCTTTCTCACA
Cav1	GCACACCAAGGAGATTGACC	TCCCTTCTGGTTCTGCAATC
Cyr61	TGCTGTAAGGTCTGCGCTAA	AGGGTCTGCCTTCTGACTGA
Vegfc	GGGAAGAAGTTCCACCATCA	TTCCAATACGATGGGACACA
Dll4	ACCTTTGGCAATGTCTCCAC	TTGGATGATGATTTGGCTGA
Col4a1	GGCCCTTCATTAGCAGGTGT	GTGAGGACCAACCGTTAGGG
Fzd4	CCAGCTGCAGTTCTTCCTTTG	GGCCAGGCAAACCCAAATTC
Tcf7l2	GGGAAACCAACGAACACAGC	GGCCGCACCAGTTATTCTGT
Tcf4	AGGTGCTCGGTGAATTTTCCT	GGCGAAAACATCGCACTGAAA
Meis2	CTCACACACCCGTACCCTTC	TGGCTCACTGCTCGATTTGA
Zeb1	GCTGGCAAGACAACGTGAAA	GGGCGCCTCAGGATAAATGA
HoxD8	GAGGCCGAGCTGGTACAATA	CAGGAGCTGCTTGTGGTCTC
Acvrl1	TGGTCACCACAGCTATCACG	CAAGTCTCCCCTGGTTAGGC
Itgb1	CGCATTGGCTTTGGCTCATT	AATGGGCTGGTGCAGTTTTG
Egfl7	GAAGAAGGCTACCCCACTTACA	TACACACTCTACGGCTGGGT
Lrp5	TTCCAACATGCTGGGTCAGG	GCCCGTTCAATGCTATGCAG
Snai2	CACATTGCCTTGTGTCTGCAA	AAGAGAAAGGCTTTTCCCCAGT
Vwf	TGCTGCCCAGAGTATGAGTG	CTTTCCTGCAGGCACAGGTA
Rhoc	GAAACAGGAGCCGGTTCGAT	CCCGAGTGGCCATCTCAAAT
Anxa2	GGGTGAAGAGGAAAGGAACC	TTGATGCTCTCCAGCATGTC
Flt1	CTGAAGCGGTCTTCTTCCGA	GTGCAAACTCCCACTTGCTG
Itga2	TGCGGCTGCTAATGCTAGTT	CCAGTAGCCAGTTGCCTTGT
Sox18	CAAACTGCCGACGAGTTGCG	ACGCTTTGCCCAGCATCTTG
Efnb2	CCAGGAATCACGGTCCAACA	TGCTAGAACCTGGATTTGGCT
Ccnd1	GATGGCGATCGTCCTGTCAT	ACAGGCCGCTACAAGAAACA
Hes5	TGAAACACAGCAAAGCCTTC	GTGCAGGGTCAGGAACTGTA
Itga2b	AGAGTGAAGGGCTGAGTCCA	GCTCCATATGCTCCCACAAT
Jag1	GACCAGAACGGCAACAAAACTTGCATGGAA	TTGGTCTCACAGAGGCACTGCCAGGGTTCA
Hes1	CGGCATTCCAAGCTAGAGAAGG	GGTAGGTCATGGCGTTGATCTG
Gata2	ACACACCACCCGATACCCACC	CCACAGGCGTTGCACACAGG
Runx1	CACTGCCTTTAACCCTCAGC	GAGGTGATGGATCCCAGGTA
Kit	GGCTGGCATCACCATCAAAAAC	AGGCACAGACACAACAGGGATAGC
Gapdh	TGTTCCTACCCCCAATGTGT	TGTGAGGGAGATGCTCAGTG
Hprt	GTTAAGCAGTACAGCCCCAAA	AGGGCATATCCAACAACAAACTT

Supplementary Table 1. List of mouse primers used for Pre Amplifications reactions and qRT-PCR

Supplementary Table 2

GO Biological Processes in Kit+_Kit- comparison					
Name	Observed events	Expected mean	Expected stddev	N	Corrected right P-Value
developmental process	171	48.6	6.90	2362	<1.0e-16
angiogenesis	42	3.95	1.97	192	<1.0e-16
regulation of cell migration	43	4.96	2.20	241	<1.0e-16
cell adhesion	62	12.3	3.47	599	<1.0e-16
organ development	66	15.3	3.87	745	<1.0e-16
cell differentiation	77	22.5	4.70	1095	<1.0e-16
heart development	32	3.97	1.97	193	<1.0e-16
regulation of cell proliferation	57	14.3	3.74	694	6.11e-16
positive regulation of cell migration	28	3.43	1.83	167	2.72e-15
regulation of cellular metabolic process	71	22.3	4.68	1085	8.77e-15
response to stimulus	205	110	10.4	5356	2.56e-14
lymph vessel development	10	0.247	0.492	12	1.02e-13
positive regulation of gene expression	56	15.7	3.92	763	1.02e-13
positive regulation of transcription, DNA-dependent	56	16.0	3.95	776	1.85e-13
vasculogenesis	17	1.36	1.15	66	1.62e-12
blood vessel morphogenesis	14	0.864	0.920	42	5.43e-12
vascular endothelial growth factor receptor signaling pathway	11	0.452	0.666	22	1.10e-11
extracellular matrix organization	20	2.37	1.52	115	2.14e-11
cellular component movement	32	7.03	2.62	342	1.31e-10
signal transduction	131	67.6	8.14	3286	1.72e-10
cell motility	31	6.79	2.58	330	2.47e-10
tube morphogenesis	20	2.86	1.67	139	6.56e-10

blood vessel development	16	1.73	1.30	84	8.98e-10
branching morphogenesis of a tube	18	2.34	1.52	114	1.34e-09
cellular response to stimulus	141	78.0	8.74	3792	2.71e-09
morphogenesis of a branching structure	18	2.45	1.55	119	2.68e-09
positive regulation of BMP signaling pathway	10	0.555	0.737	27	3.95e-09
semaphorin-plexin signaling pathway	8	0.288	0.531	14	4.03e-09
cell-cell adhesion	25	5.20	2.26	253	7.51e-09
actin filament-based process	22	4.26	2.04	207	2.03e-08
cytoskeleton organization	31	8.33	2.86	405	2.79e-08
embryo development	26	6.07	2.44	295	3.21e-08
positive regulation of MAPK cascade	13	1.36	1.15	66	3.22e-08
chemotaxis	23	4.83	2.18	235	3.79e-08
axon guidance	17	2.61	1.60	127	4.71e-08
lymphangiogenesis	7	0.267	0.512	13	8.48e-08

Supplementary Table 2- List of selected enriched GO Biological Processes in Kit+_Kit- comparison. Terms sorted by corrected right p-value. p-value scale. Multiple test correction Benjamini Hochberg FDR.

Supplementary Table 3

GO Biological Processes in Kit-J_Kit- comparison					
Name	Observed events	Expected mean	Expected stddev	N	Corrected right P-Value
developmental process	95	26.7	5.14	2362	<1.0e-16
angiogenesis	26	2.17	1.47	192	<1.0e-16
regulation of cell migration	28	2.73	1.64	241	<1.0e-16
cell adhesion	37	6.78	2.59	599	1.33e-13
regulation of signal transduction	42	10.5	3.22	924	2.87e-11
regulation of cell proliferation	37	7.86	2.79	694	7.93e-12
regulation of metabolic process	51	16.2	4.00	1432	5.78e-10
anatomical structure formation involved in morphogenesis	22	3.23	1.79	285	6.62e-10
organ development	35	8.43	2.89	745	7.81e-10
cell differentiation	43	12.4	3.50	1095	1.05e-09
response to external stimulus	24	4.08	2.01	360	1.30e-09
vasculogenesis	12	0.747	0.860	66	1.71e-09
lymph vessel development	7	0.136	0.366	12	2.34e-09
blood vessel morphogenesis	10	0.476	0.686	42	4.32e-09
vascular endothelial growth factor receptor signaling pathway	8	0.249	0.496	22	7.74e-09
response to stimulus	116	60.6	7.74	5356	1.26e-08

morphogenesis of a branching structure	14	1.35	1.15	119	1.10e-08
positive regulation of MAPK cascade	11	0.747	0.860	66	2.10e-08
cellular component movement	21	3.87	1.96	342	5.76e-08
branching morphogenesis of a tube	13	1.29	1.13	114	5.81e-08
tube morphogenesis	14	1.57	1.25	139	6.66e-08
heart development	16	2.19	1.47	193	7.50e-08
cell motility	20	3.74	1.92	330	1.60e-07
actin filament-based process	16	2.34	1.52	207	1.85e-07
response to vitamin	13	1.45	1.20	128	2.01e-07
blood vessel development	11	0.951	0.970	84	2.06e-07
response to mechanical stimulus	11	0.962	0.975	85	2.28e-07
positive regulation of cellular biosynthetic process	31	8.86	2.96	783	2.39e-07
semaphorin-plexin signaling pathway	6	0.159	0.396	14	3.29e-07
extracellular matrix organization	12	1.30	1.13	115	5.01e-07
regulation of endothelial cell proliferation	8	0.532	0.725	47	2.58e-06
response to extracellular stimulus	17	3.31	1.81	292	2.75e-06

Supplementary Table 3, - List of selected enriched GO Biological Processes in Kit-J_Kit- comparison. Terms sorted by corrected right p-value. p-value scale. Multiple test correction Benjamini Hochberg FDR.
